# Ebola virus VP30 and nucleoprotein interactions modulate viral RNA synthesis

**DOI:** 10.1038/ncomms15576

**Published:** 2017-06-08

**Authors:** Wei Xu, Priya Luthra, Chao Wu, Jyoti Batra, Daisy W. Leung, Christopher F. Basler, Gaya K. Amarasinghe

**Affiliations:** 1 Department of Pathology and Immunology, Washington University School of Medicine, St Louis, Missouri 63105, USA; 2 Center for Microbial Pathogenesis, Institute for Biomedical Sciences, Georgia State University, Atlanta, Georgia 30303, USA

## Abstract

Ebola virus (EBOV) is an enveloped negative-sense RNA virus that causes sporadic outbreaks with high case fatality rates. Ebola viral protein 30 (eVP30) plays a critical role in EBOV transcription initiation at the nucleoprotein (eNP) gene, with additional roles in the replication cycle such as viral assembly. However, the mechanistic basis for how eVP30 functions during the virus replication cycle is currently unclear. Here we define a key interaction between eVP30 and a peptide derived from eNP that is important to facilitate interactions leading to the recognition of the RNA template. We present crystal structures of the eVP30 C-terminus in complex with this eNP peptide. Functional analyses of the eVP30–eNP interface identify residues that are critical for viral RNA synthesis. Altogether, these results support a model where the eVP30–eNP interaction plays a critical role in transcription initiation and provides a novel target for the development of antiviral therapy.

## Abstract (web summary)

Ebola virus (EBOV) VP30 is a multifunctional protein that plays a role in transcription, but molecular details remain unknown. Here, using X-ray crystallography and minigenome assays, Xu *et al*. define the interaction between VP30 and a portion of NP that is critical for optimal EBOV RNA synthesis.

Ebolavirus, Marburgvirus, and Cuevavirus are members of the *Filoviridae* family. Members of the Ebolavirus genus and the Marburgvirus genus cause rare outbreaks with high case fatality rates^1^,^2^. Ebola virus (EBOV), Sudan virus, Taï Forest virus, Bundibugyo virus, and Reston virus are species within the genus Ebolavirus^3^. The recent outbreak in West Africa, which also imported cases to non-West African countries, serves as a reminder of the potential impact of filovirus infections on global health (http://www.who.int/csr/disease/ebola/en). While recent efforts to develop countermeasures that target filoviruses are starting to yield candidate therapeutics, our knowledge of the basic biology of the virus, including key virus–virus and host–virus interactions remain incompletely defined^4^,^5^. Moreover, it is currently unclear if the current candidate therapeutics will prove safe and efficacious in humans, and therefore the need for antifilovirals and novel targets remain.

Viral protein 30 (VP30) proteins are present in all filoviruses and perform several important functions, but homologues are lacking in other negative-sense RNA viruses^6^,^7^,^8^,^9^,^10^,^11^. VP30 proteins are approximately 30 kDa in size and are phosphoproteins, where the phosphorylation state is thought to impact some but not all VP30-mediated functions^10^,^12^. Additionally, VP30 binds RNA in the presence of zinc^7^ and associates with the viral RNA synthesis machinery, which includes nucleoprotein (NP), VP35, and large (L) protein that contains the enzymatic activities^9^,^12^,^13^,^14^,^15^. EBOV VP30 (eVP30) is critical for viral transcription (mRNA synthesis), because it is required for initiation at EBOV NP (eNP), the first gene of the seven gene genome^16^. eVP30 has also been implicated in regulation of co-transcriptional editing of viral glycoprotein mRNAs and in modulation of viral transcription reinitiation^11^,^17^. In contrast to the studies on eVP30, there does not appear to be a requirement for VP30 in Marburg virus (MARV) transcription initiation, at least when measured using model viral RNA templates. Nonetheless, attempts to generate replication-competent VP30 deletion MARVs were unsuccessful, suggesting an essential role in virus growth^18^ and potentially additional roles for VP30 in MARV and in EBOV. eVP30 has been shown to interact with eVP35 and with eNP, and these interactions may modulate viral transcription and incorporation of viral genomic RNAs into new viral particles^12^,^15^. An additional report suggested that VP30 interacts with the viral polymerase L^13^.

The studies described above collectively point to significant roles played by eVP30 through various intermolecular interactions, which likely occur at different stages of the virus replication and infection cycles. While these interactions have been previously identified, we currently lack a molecular description of them. Importantly, we do not know how these interactions regulate function. Defining mechanisms by which eVP30 modulates viral RNA synthesis and other aspects of viral replication also has the potential to identify novel therapeutic targets. Because the interaction of eVP30 with eNP may play roles in viral transcription and assembly, we investigated here how eVP30 and eNP interact with each other, leading to a biochemical and structural definition of the binding interface. We demonstrate that eVP30 binds directly to eNP. We show that mutation of a subset of VP30 residues important for NP binding results in loss of RNA synthesis, while mutation at other sites do not. Moreover, mutation of multiple residues on NP involved in VP30 binding also results in loss of function. We also note that the significance of this interaction depends on a specific secondary structure of the RNA template^16^. This stem loop, which was previously identified as an eVP30 interactor, dictates whether eVP30/eNP interaction is required or dispensable for minigenome (MG) activity. Consistent with this observation, in our modified MG assay (MGA), we show that this specific stem loop that interacts with eVP30 may control virus replication and transcription in infected cells. Collectively, our data reveal new insights that address the enigmatic nature of VP30 function and, importantly, define novel targets for therapeutic development, including eVP30–eVP30 and eVP30–eNP interaction sites.

## Results

### A peptide derived from eNP binds VP30

**Figure 1:**
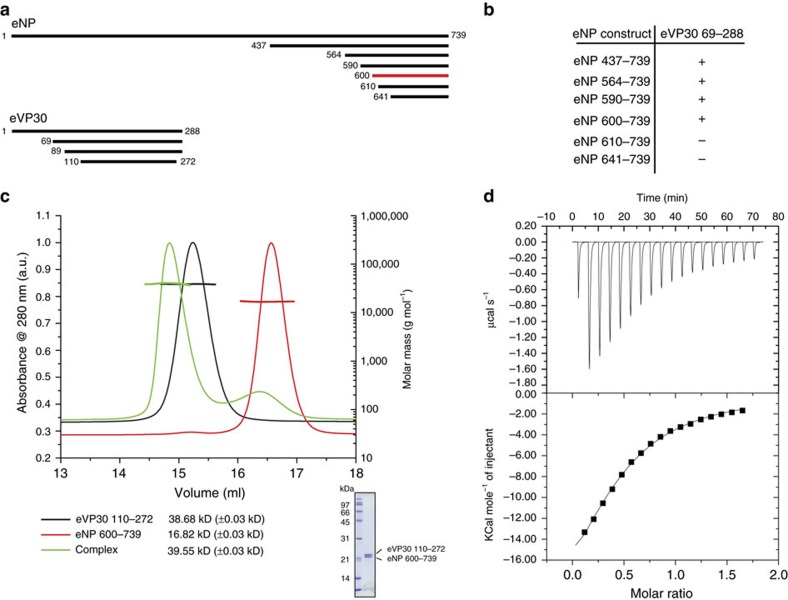
eVP30 binds eNP with high specificity. (**a**) Constructs of eNP (top) and eVP30 (bottom) used in this study. (**b**) Summary of *in vitro* pull-down-based binding results for a series of truncation constructs for eNP. +, binding; −, no binding. (**c**) Size exclusion chromatography coupled to multi-angle light scattering results for eVP30_110–272_ (black; molecular weight (MW) 38.7±0.030 kDa), eNP_600–739_ (red; MW 16.8±0.030 kDa) and eVP30_110–272_-eNP_600–739_ complex (green; MW 39.6±0.030 kDa). The calculated MW for monomeric eVP30_110–272_, dimeric eVP30_110–272_ and eNP_600–739_ are 18.7, 37.3 and 17.03 kDa, respectively. The calculated MW for a 1:1 complex of eVP30_110–272_–eNP_600–739_ is 35.7 kDa. (**d**) Representative ITC binding isotherm for the 1:1 complex between eVP30_110–272_ and eNP_600–739_. *K*_D_=3.75±1.7 μM. (Also see Supplementary Fig. 1). Error represents s.d.

In order to gain molecular mechanistic insight into how eVP30/eNP interaction promotes viral RNA synthesis, we generated a series of recombinant eVP30/eNP truncation constructs (Fig. 1a; Supplementary Fig. 1). Initially, we tested eVP30/eNP protein–protein interactions by *in vitro* pull-down assays, which revealed that a region encompassing residues 600–610 in eNP is critical for eVP30 interactions (Fig. 1b; Supplementary Fig. 2a–e). This region of eNP was absent in the solved crystal structures of the eNP C-terminus (residues 642–739; PDB 4QB0, 4QAZ (ref. 19), Tai Forest NP (tNP; PDB: 5E2X) and Bundibugyo virus NP C-terminus (residues 642–739; PDB 5DSD)^20^ and therefore represents a new finding. Next, we optimized the crystallization by varying constructs (eNP_600–739_ and eVP30_110–272_) and characterized this interaction by size-exclusion chromatography coupled to multi-angle light scattering. These results indicate that eVP30_110–272_ behaves as a dimer while eNP_600–739_ is a monomer in solution (Fig. 1c). Upon mixing, eNP_600–739_ and eVP30_110–272_ form a 1:1 heterodimer complex (Fig. 1c; Supplementary Fig. 2f–h). Consistent with this, isothermal titration calorimetry (ITC) experiments revealed that eNP and eVP30 bind with 3.75±1.7 μM affinity and 0.8±0.2 stoichiometry, with ± denoting s.d. of triplicate replicates (Fig. 1d). Together, the size-exclusion chromatography coupled to multi-angle light scattering and ITC data suggest that complex formation leads to loss of VP30 dimerization. Of note, the eVP30_110–272_ construct lacks the major dimerization site defined previously^21^, suggesting that additional regions within VP30 are involved in oligomerization and regulation of VP30 function^12^.

**Figure 2:**
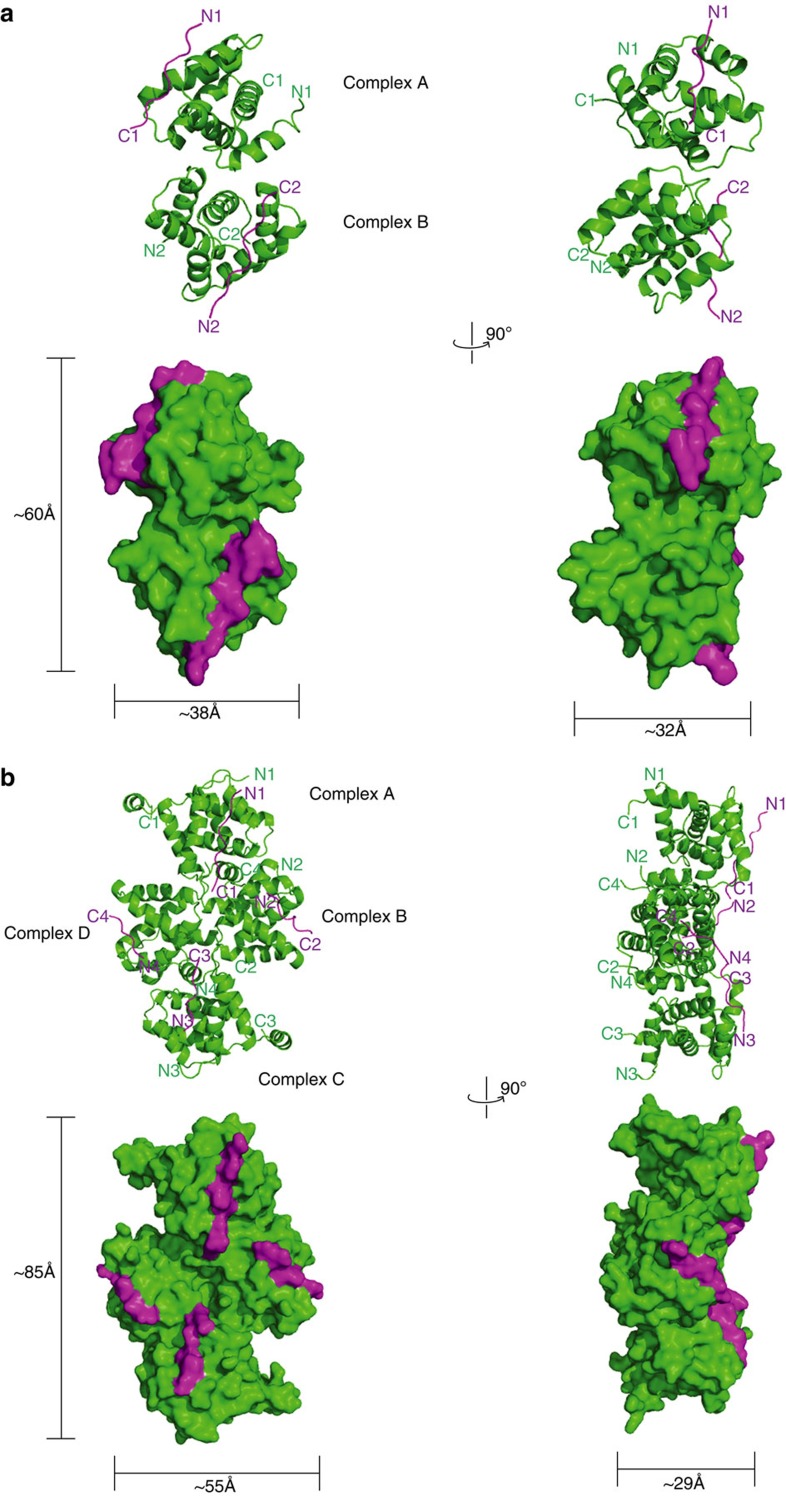
A peptide derived from eNP binds eVP30 using the same interface despite different VP30 complexes in the asymmetric unit. Cartoon (top) and surface (bottom) representations of the following complexes are shown: (**a**) 2:2 ratio of eVP30 (green) and eNP (magenta). (**b**) 4:4 ratio of eVP30 (green) and eNP (magenta). (Also see Supplementary Fig. 1).

We generated protein crystals for structure determination using the purified eVP30_110–272_/eNP_600–739_ complex. Initial low-resolution diffracting crystals were optimized by *in situ* proteolysis and resulted in several well-diffracting crystals. The two highest-resolution data crystal forms were solved by molecular replacement using the Reston virus VP30 structure (PDB 3V7O) as the search model and were refined iteratively are described here (Fig. 2 and Table 1). In the structures, we observe eNP residues 602–612 with residues 140–252 (5VAP) and residues 140–266 of eVP30 (5VAO). Comparisons between molecules within each asymmetric unit (two complexes in 5VAP and four complexes in 5VAO) as well as comparisons of structures across different space groups revealed minimal conformational perturbations. For example, heavy atom pairwise root-mean-square deviation for eVP30 (residues 140–266) ranges from 0.4 to 1.1 Å, while similar comparisons for eNP result in a pairwise root-mean-square deviation of 0.3–1.3 Å. In contrast to eVP30/eNP contacts, which are largely similar even though the structures belong to different asymmetric units, the ternary configurations of eVP30 suggest that the eVP30/eNP interaction may not impact eVP30–eVP30 interactions (Fig. 2). However, it is important to note that our *in vitro* data suggest that the interaction between eNP and eVP30 can impact the oligomerization state of eVP30 in solution. These differences may play a critical role in a context-dependent manner during virus replication and assembly. Comparison of the eVP30 structure for residues 142–266 between VP30-bound and -free show limited structural changes (Supplementary Fig. 3). Given the overall agreement among the eVP30/eNP 1:1 complexes, we used complex A from 5VAP as representative structures for analyses and discussions below.

### Interface between eVP30 and eNP is highly conserved

**Figure 3:**
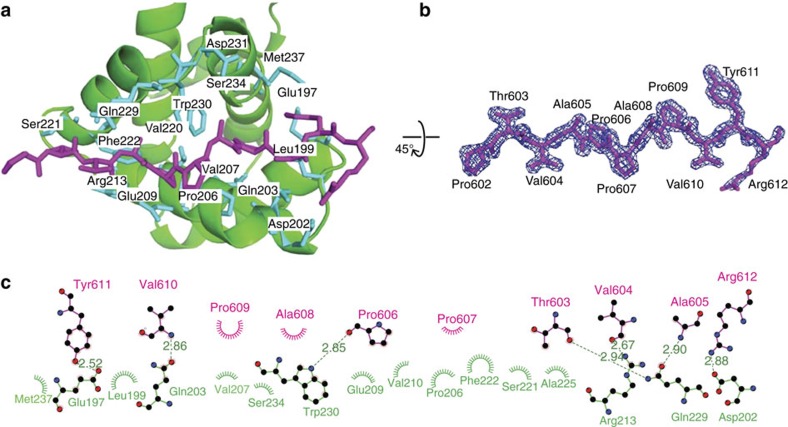
eNP binds within a highly conserved narrow groove within eVP30 via a combination of hydrophobic and electrostatic interactions. (**a**) eVP30 residues (green) involved in interactions with eNP are shown in stick representation (blue) and labelled. The eNP peptide is shown in stick representation (purple). (**b**) Stick representation of the eNP peptide residues 602–612 (purple) with a 2*σ* electron density map (sigma-weighted 2Fo-Fc; blue mesh). Orientation is 45° rotated from the orientation in **a**. (**c**) LigPlot^+^ representation of the protein–protein interactions between eVP30 (green) and eNP (purple). Protein side chains are shown as ball and sticks. Hydrogen bonds are shown as green dotted lines. Spoked arcs represent non-bonded contacts and the length of the arc represents the extent of the interaction. (Also see Supplementary Fig. 3).

Residues from eNP that span 600–612 are highly conserved not only across all ebolavirus genus members but also marburgvirus as well (Supplementary Fig. 1a). Sequence analysis of human proteins suggests that this 13 residue sequence observed in our structure is not a common eukaryotic sequence. Moreover, given the structural requirements that are critical for binding, the likelihood of a host factor mimicking the eNP peptide sequence is low. Although PPXP motifs typically form a left-handed turn, in the eNP/eVP30 complex structures, the conformation of this motif does not form the expected polyproline type II helix. Instead, these highly conserved eNP residues make extensive contacts with eVP30 residues in an extended conformation (Fig. 3a). Although the binding pocket is somewhat shallow, the interaction site displays a shape complementarity (Sc) score of 0.6, which is considered significant. For comparison, antibody–antigen complexes have Sc scores of around 0.72. Moreover, examination of the electron density of the unbiased map (Supplementary Fig. 4a) or the final refined map (Fig. 3b) shows clear density for the secondary structure with *B*-factors comparable to nearby residues of eVP30, further supporting the relevance of the crystallographically identified interface. Analysis of the interface reveals that there are seven hydrogen bonds between eVP30 and eNP (Fig. 3c) and a number of hydrophobic contacts, which make up much of the observed total of 1,923 Å^2^ buried surface area.

### Select interface residues are important for RNA synthesis

**Figure 4:**
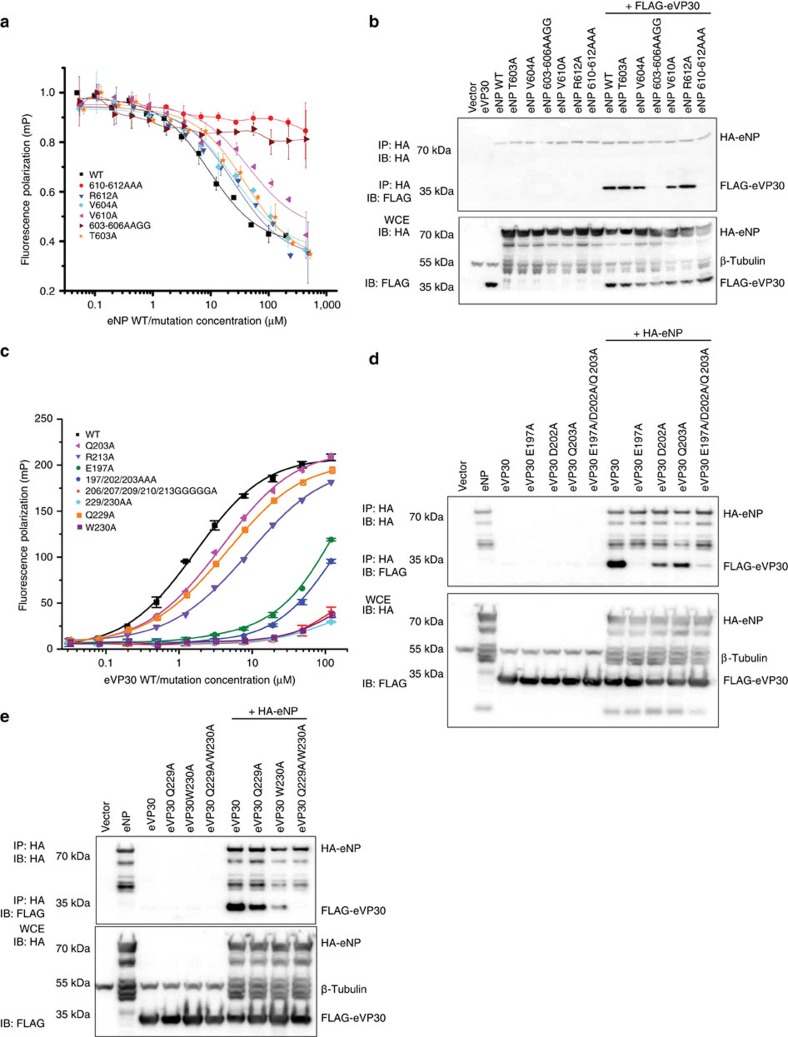
Effects of eNP interface residue mutations on eVP30 binding. (**a**) FP competition assay measuring binding between eNP_590–739_ WT and mutants and FITC-labelled eVP30BP. Error bars represent s.d. of at least three experiments. (**b**) Immunoprecipitation (top) and corresponding western blots (bottom) of mutant HA-eNP with WT Flag-eVP30. (**c**) FITC–eVP30BP binding with WT or mutant eVP30_110–272_ measured by FP. Error bars represent s.d. of at least three experiments. (**d**,**e**) Co-immunoprecipitation of WT HA-eNP full length with WT or mutant FLAG-eVP30 full length. IP, immunoprecipitation; IB, immunoblot; WCE, whole-cell extract; E, empty vector.

In order to experimentally validate the interface between eVP30 and eNP, we developed a fluorescence polarization assay (FPA) to evaluate effects of mutating interface residues on eVP30 and eNP binding. Peptides corresponding to eNP residues 600–615, which we termed EBOV VP30-binding peptide (eVP30BP), were conjugated to fluorescein isothiocyanate (FITC-eVP30BP). FPA was performed by increasing eVP30 concentration (Supplementary Fig. 4b) in the presence of FITC-eVP30BP to monitor the fluorescence decrease as a result of the slower tumbling of FITC-eVP30BP after binding to eVP30. In competition FPA, constant concentrations of FITC-eVP30BP and eVP30_110–272_ were incubated with increasing concentrations of unlabelled eVP30BP competitor (Supplementary Fig. 4c). Both types of experiments display a high dynamic range of >100 and >50 arbitrary units, respectively. Using these FPAs, we tested mutants of eNP and eVP30 and identified a number of amino acid residues that showed in a minor decrease in binding when individually mutated (Fig. 4a). eNP T603A, V604A, V610A and R612A had little or no impact on eVP30 binding while multi-residue mutants (603-606AAGG and 610-612AAA) resulted in a near complete loss of binding to eVP30 wild type (WT), suggesting that NP contributes a large number of redundant contacts to eVP30 binding. Consistent with this finding, co-immunoprecipitation of FLAG-eVP30 with HA-NP WT or mutants revealed results that correspond to our *in vitro* FPA results (Fig. 4b). When we tested eVP30 mutants, we found that some single eVP30 mutations, but not others, resulted in loss of binding to eNP WT. For example, eVP30 Q203A or R213A or Q229A did not result in loss of binding to eNP WT in the FPA, whereas eVP30 E197A and W230A resulted in a near complete loss of binding (Fig. 4c–e).

**Figure 5:**
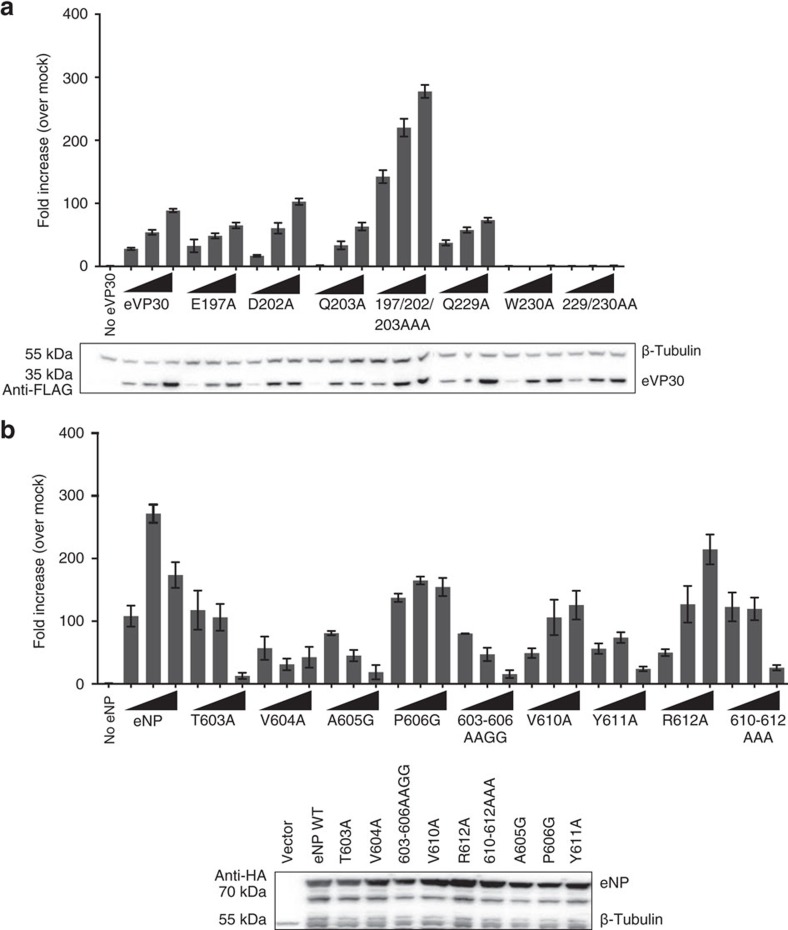
Interaction between eVP30 and eNP observed in the crystal structure is not strictly required for viral RNA synthesis. MGA was performed using (**a**) WT and select interface mutants of eVP30 (50, 100, 200 ng) and (**b**) WT and select interface mutants of eNP (125, 250, 500 ng) (western blots represent the expression of eNP at 500 ng). Representative western blots are shown. Errors represent s.d. of at least three experiments.

We next assessed the eVP30/eNP interface using an Ebola minigenome assay (MGA), which reports on viral RNA synthesis^22^. We observed that mutations in eVP30, such as Q203A and Q229A (which slightly affect binding) or E197A (which largely abrogates binding between eVP30/eNP), have limited impact in the MG activity (Fig. 5a). Furthermore, this was also true for eNP mutants when tested in the MGA. The multiple eNP residue mutants that completely abrogated binding to eVP30 such as 603–606 AAGG and 610–612 AAA (Fig. 4b) also showed less MG activity upon titration of eNP residue mutants (Fig. 5b). Interestingly, the individual residue mutants of eNP, which only minimally affected binding, had little effect on MG activity as well. These results suggest that a high-affinity interaction at the site we observe in our crystal structure between eVP30 and eNP via the eVP30BP is not strictly required for viral RNA synthesis. Moreover, our data suggest that even minimal binding between eVP30-eNP is sufficient for this interaction to function in viral RNA synthesis. However, our data show that the binding site identified in our structure, which includes residues such as W230, results in complete loss of binding and corresponding loss of activity in viral RNA synthesis. Although the direct impact of W230 in viral RNA synthesis is unclear, given the importance of eNP/eVP30 interaction defined in the crystal structure, it is likely that sustained interaction is required for viral replication. However, an alternative possibility is that the molecule may lose structure due to the loss of hydrophobic contact and stability.

### eVP30 and eNP interaction recognizes RNA stem loop

**Figure 6:**
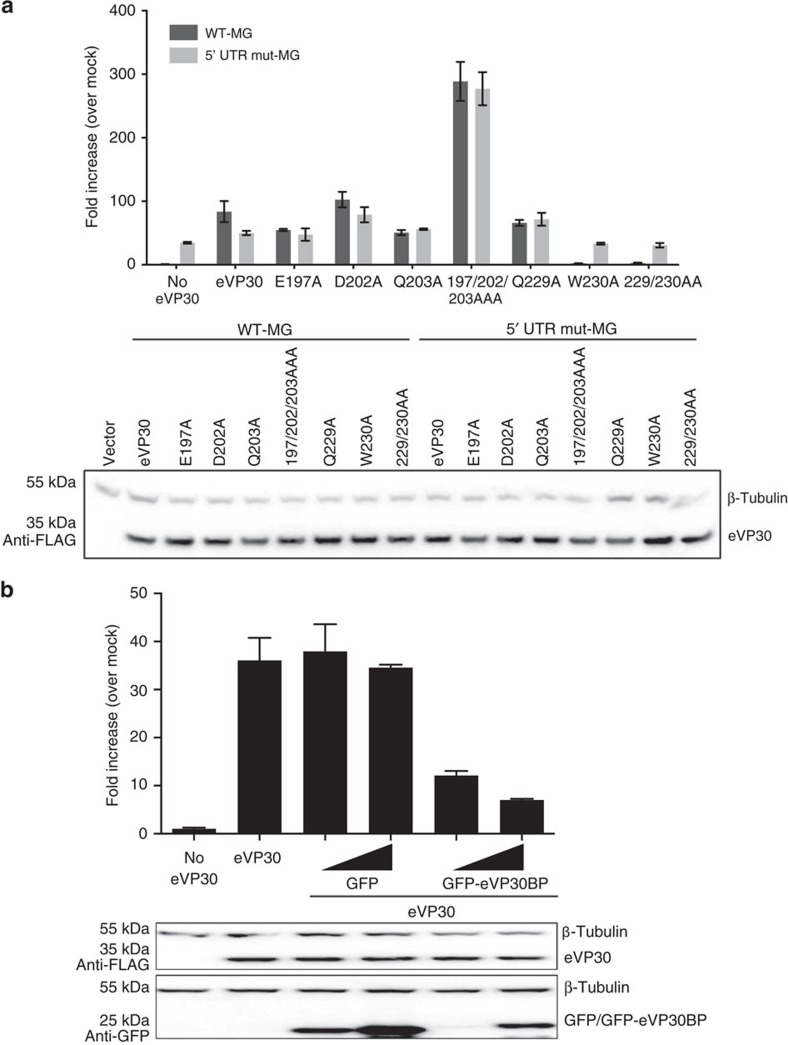
Additional residues at the eVP30 and eNP interface may be important for viral RNA synthesis and transcription. (**a**) VP30 interface mutants with WT (dark grey bar) or stem-loop 5′UTR mutant MG template (light grey bar). (**b**) The MG assay was performed in the presence of either GFP control plasmid or plasmid consisting of GFP-eVP30BP (used at 50, 500 ng). The effect of GFP-eVP30BP (500 ng) was examined on the WT-MG. Representative western blots are shown. Errors represent s.d. of at least three experiments.

To further determine the relevance of the eNP binding to eVP30 in viral RNA synthesis, we performed experiments to compare results from WT and mutant MG templates. The mutant MG has a mutation in a stem-loop RNA structure that corresponds to one in the 5′UTR (untranslated region) region of eNP, which renders EBOV transcription VP30 dependent^16^. The resulting data, consistent with previously published studies^16^, revealed that the mutant MG can function in a VP30-independent manner (Supplementary Fig. 4d). Viral RNA synthesis was assessed with either eVP30 or eVP30 mutants using either WT or mutant MG assays. From this data, we find that mutant MG activity was comparable to the WT MG activity for all eVP30 mutants (Fig. 6a). In addition, we observe that eVP30 mutants that were significantly inhibited in the WT MG assay, including the W230A mutant (and Q229A/W230A), were functional in the mutant MG assay (Figs 5a and 6a). These results suggest that these mutants that lack activity with the WT MG are defective in viral transcription initiation. Given that some mutations within the interface do lose activity, this suggests that the residues involved in the interaction play an additional role in viral transcription.

**Figure 7:**
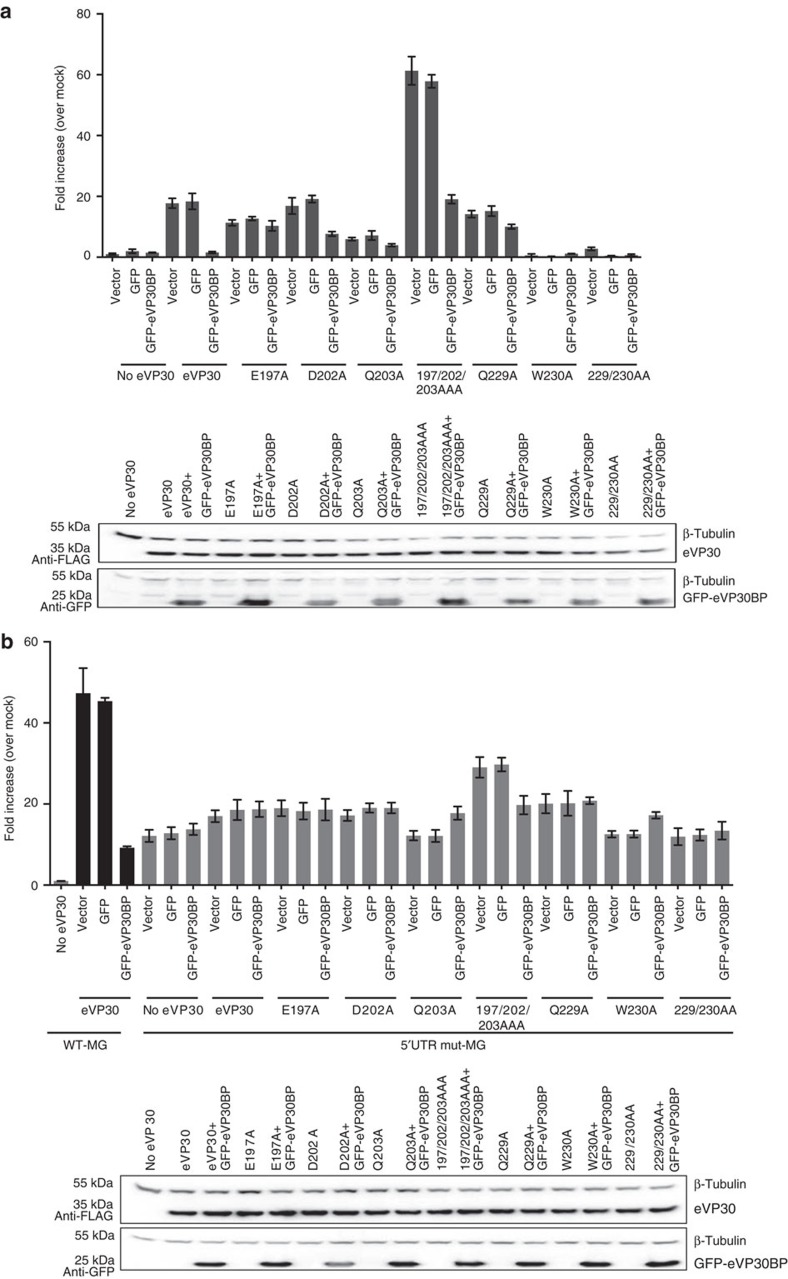
eVP30BP inhibits VP30-dependent viral RNA synthesis. (**a**) WT MG or 5′UTR mutant MG template (**b**) in the presence of VP30 interface mutants. The corresponding western blots for each MG assay is shown below each chart. (Also see Supplementary Fig. 4). The MG data and representative western blots are shown. Errors represent s.d. of at least three experiments.

We further evaluated whether this highly conserved eNP region (600–615 residues) that makes contacts with eVP30 (eVP30-binding peptide) has the ability to affect viral RNA synthesis. We generated a green fluorescent protein (GFP) fusion with the eNP 600–615 region (GFP-eVP30BP) and tested its role in the MG assay. These results, shown in Fig. 6b, revealed that GFP-eVP30BP but not GFP alone resulted in dose-dependent inhibition of MG activity. This result suggests that the eVP30BP from eNP makes contact with eVP30 in the context of the viral RNA polymerase complex and has the potential to inhibit viral RNA synthesis. We also tested the effect of GFP-eVP30BP in the presence of eVP30 mutants that are unable to bind to NP (Fig. 7a). The effect of this peptide on MG activity correlated to the binding affinities of each eVP30 residues to eNP. For example, the MG activity with WT eVP30 was inhibited in the presence of peptide. MG activity with single eVP30 mutants that largely bind to eNP (D202A, Q203A or Q229A) were also inhibited by peptide. However, the peptide failed to inhibit MG activity with eVP30 E197A, which does not bind to eNP. We also evaluated the effect of peptide using the 5′UTR mutant MG construct (Fig. 7b). The presence of peptide had no effect on activity with this mutant MG template, suggesting that this peptide inhibits VP30-dependent viral RNA synthesis. Cumulatively, these results indicate that there are eVP30/eNP-dependent and -independent components of Ebola viral RNA synthesis. The eVP30–eNP component can be targeted by the eNP peptide in the context of the viral polymerase complex and inhibit viral synthesis.

## Discussion

Our results described above define how eVP30 interactions with eNP impact EBOV RNA synthesis and identify novel targets for virus-directed therapeutic development. Specifically, we use an eNP-derived peptide and show that the VP30-binding site can be a target for peptide or peptide-mimic therapeutics as well as its utility for high-throughput assay. Our structural and cell biological studies, which are highlighted in Supplementary Table 1, provide a molecular mechanism by which the interaction between eVP30 and eNP promotes RNA synthesis of EBOV MG activity. While we identify specific molecular interfaces, which were validated using a novel competition assay in order to demonstrate the relevance of our studies and to define a key conserved interface, we also show that the regulatory interactions between eVP30 and eNP are more complex than originally appreciated. Our studies also show that eVP30/eNP interaction can impact the oligomeric state of eVP30, which may be functionally relevant. Importantly, we show that a subset of the eVP30/eNP interaction defined by our study is critical for viral RNA synthesis in a RNA secondary structure-dependent manner, while others may be dispensable. It is important to note that our results presented here point to the significance of eVP30/eNP in RNA synthesis initiation, but previous studies indicate that eVP30 may also play a role as a transcriptional activator. Both of these activities likely depend on eVP30/eNP interactions, which further enhance the significance to viral RNA synthesis. A recent study of the Ebola viral VP30/NP concluded that this interaction is as a regulator of viral RNA synthesis^23^. This observation is consistent with the results reported here. Together, these studies highlight the significance of this VP30/NP interaction, and in particular, we show that this interaction, at least in part, is significant for recognition of a critical stem loop in the viral UTR. Further efforts to examine this interaction for therapeutic development are likely to result in new insights into the complex molecular mechanism of filoviral RNA synthesis.

## Methods

### Constructs

Full-length Ebola Zaire VP30 (eVP30) gene (GenBank ID KY425656.1) was used as a template to generate PCR products for eVP30_69–288_, eVP30_89–288_ and eVP30_110–272_ constructs that were ligated into a modified pET vector (Novagen). Resulting vectors were verified by sequencing before use. EBOV eNP_437–739_, eNP_564–739_, eNP_590–739_, eNP_600–739_, eNP_610–739_ and eNP_641–739_ were cloned similarly.

### Protein expression and purification

All eVP30 and eNP proteins were expressed as maltose-binding protein (MBP) fusion proteins in BL21(DE3) *E. coli* cells (Novagen) in Lysogeny broth media. Protein expression was induced at an optical density of 0.6 with 0.5 mM isopropylthiogalactoside and grown for 12–15 h at 18 °C. Cells were harvested and resuspended in lysis buffer containing 25 mM sodium phosphate pH 7.5, 250 mM NaCl, 20 mM imidazole and 5 mM 2-mecaptoethanol. Then cells were lysed using an EmulsiFlex-C5 homogenizer (Avestin) and clarified by centrifugation at 30,000*g* at 4 °C for 30 min. eVP30 and eNP constructs were purified using a series of affinity and ion exchange chromatographic columns. The MBP tag was cleaved using TEV protease prior to a final application on a size-exclusion column. The purity of the samples was determined by SDS–PAGE. For eVP30/eNP complexes, purified eVP30 and eNP proteins were mixed in a 1:1.1 ratio, purified on a Superdex 75 column (GE Healthcare) and concentrated to 20 mg ml^−1^.

### *In vitro* pull-down assays

Amylose resin was pre-equilibrated with buffer (20 mM Tris, pH 7.5, 150 mM NaCl, 5 mM 2-mecaptoethanol) prior to the addition of lysate containing recombinantly expressed MBP-tagged proteins at 4 °C. Resin was incubated for 10 min, followed by washes and subsequent resuspension in buffer. Cell lysate of cells expressing either WT or mutant versions of eNP and eVP30 were applied to the resin and allowed to incubate for 20 min, prior to washes and final resuspension in buffer. Samples were taken at each step and visualized by Coomassie blue staining of SDS–PAGE.

### Isothermal titration calorimetry

Quantitative analysis of eVP30/eNP complex binding was performed on a VP-isothermal titration calorimeter (VP-ITC) (Microcal). Protein samples were dialysed against 500 ml of buffer containing 10 mM HEPES (pH 7.0), 150 mM NaCl and 2 mM tris(2-carboxyethyl)phosphine for 12 h at 25 °C. Titrations were set up with 50–100 μM protein in the syringe and 4–10 μM protein in the cell. ITC titrations were carried out using a reference power of 4 μcal s^−1^. The resulting ITC data were processed and fit to a one-site-binding model to determine *n* (number of binding sites) and *K*_D_ (dissociation constant) using the ORIGIN 7.0 software. All experiments were performed at least in duplicate.

### Protein crystallization

The initial crystallization condition was obtained by screening Hampton Research kits using the vapour diffusion sitting drop method. In all, 20 mg ml^−1^ of eVP30_110–272_/eNP_600–739_ complex was mixed with trypsin in a 1:2,000 ratio (trypsin: eVP30_110–272_/eNP_600–739_ complex). From the initial primary screens, we observed about 10 conditions in which protein crystals appeared after 1 month and these crystals diffracted to 5–6 Å. Next, we used initial hits as seeds using homemade optimization grids in order to obtain crystals that diffracted to 1.85–3.8 Å. The final data sets came from the following growth conditions: 0.2 M potassium phosphate monobasic and 20% PEG3350 for data 5VAP, and 0.2 M calcium chloride dehydrate and 20% w/v PEG3350 for data 5VAO. Crystals appeared between 1 and 3 months at 20 °C and growth continued for another 2–6 months upon initial appearance. Crystals were vitrified in a solution containing the mother liquor plus 25% glycerol by plunge freezing in liquid nitrogen. Protease concentrations ranged from 1:10 to 1:500 protease to viral protein complex concentration (w/w).

### Data collection and structure determination

Crystallographic data were collected at Advanced Photon Source beamline 19ID (Argonne National Laboratory). Over 200 images of 0.5 degree oscillation were collected at a crystal-to-detector distance of 300 mm with an attenuation of 3. Resulting images were processed by HKL3000 (refs 24, 25). Molecular replacement was carried out with Reston virus VP30 structure (PDB ID: 3V7O) as a search model with Phaser^26^. The eNP residues were built by three cycles of Bucanneer^27^ as implemented within CCP4 (ref. 28), followed by subsequent manual model building in COOT^29^. Refinement was carried out with REFMAC5 (ref. 30). Structure quality was assessed with MolProbity^31^.

### Fluorescence polarization assays

FPA was performed on a Cytation5 plate reader (BioTek) operating on the Gen5 software. Excitation and emission wavelengths were set to 485 and 528 nm, respectively, with a band pass of 20 nm. Read height and *G* factor were set to 8.5 mm and 1.26 using the autogain function. FITC-labelled eVP30BP peptide at a final concentration of 0.5 μM was loaded onto eVP30_110–272_ samples (10 mM HEPES (pH 7.0), 150 mM NaCl and 2 mM tris(2-carboxyethyl)phosphine) at concentrations ranging from 120 to 0.031 μM with 2.5-fold dilution in a 96-well plate. After 10 min of incubation, FP signals were read. FP values were then plotted against eVP30_110–272_ concentrations, and the dissociation constant, *K*_D_, was fitted using the following equation for each mutant:


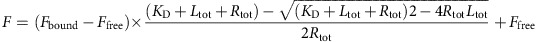


where *F*_bound_ is the FP when FITC-eVP30BP is saturated with eVP30 WT or mutants, *F*_free_ the FP of free FITC-eVP30BP, *K*_D_ dissociation constant, *L*_tot_ the total ligand concentration and *R*_tot_ the total receptor concentration.

For competition experiments, FITC-labelled eVP30BP peptide at a final concentration of 0.125 μM was mixed with 1 μM of VP30_110–272_ and eNP_590–739_ WT or mutants were added at concentrations ranging from 500 to 0.05 μM with two-fold dilution in a 96-well plate. After 20 min of incubation, FP signals were read. FP values were then plotted against eNP WT or mutant concentrations, and the dissociation constant, *K*_D_, was fitted using the following equation for each mutant:


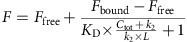


where *F*_free_ is FP of free FITC-eVP30BP, *F*_bound_ is FP of FITC-eVP30BP saturated with eVP30_110–272_, *K*_D_ is the dissociation constant of eVP30_110–272_ with FITC-eVP30BP, *C*_tot_ is the concentration of added competitor protein, *k*_2_ is the dissociation constant of eNP_590–719_ with eVP30_110–272_ and *L* is concentration of eNP_590–739_ WT or mutants.

### Co-immunoprecipitation assays

Twenty-four hours post-transfection with the indicated plasmids, HEK293T cells (ATCC, CRL-3,216) were lysed in NP-40 lysis buffer (50 mM Tris [pH 7.5], 280 mM NaCl, 0.5% Nonidet P-40, 0.2 mM EDTA, 2 mM EGTA, 10% glycerol, protease inhibitor (cOmplete; Roche)) and phosphatase inhibitor (PhosSTOP; Roche). Anti-HA magnetic beads (Sigma-Aldrich) were incubated with lysates for 1 h at 4 °C, washed five times in NP-40 lysis buffer and eluted using either HA peptide (Sigma-Aldrich). Precipitates and whole-cell lysates were analysed by western blot.

### MG assay and mutant-MG assay

HEK293T cells were transfected using Lipofectamine 2000 (Invitrogen) with plasmids encoding viral proteins: pCAGGS-L, -VP35, -NP or NP mutant constructs, -VP30, or VP30 mutant constructs along with pCAGGS-T7 polymerase^32^,^33^ and pTM1 MG reporter plasmid^34^ that encodes Renilla reporter gene flanked by *cis*-acting regulatory sequences from virus. pCAGGS plasmid is available through Addgene (https://www.addgene.org/) and the modified plasmids containing Ebola proteins are available through the authors. Some MG assays was also performed using a mutant MG reporter construct (mut MG) in which the stem-loop structure in the highly conserved transcription start signal of the NP gene (5′UTR) was disrupted where VP30 binds^16^,^22^. A constitutively active firefly plasmid (Promega) was also transfected to assess the transfection efficiency. Forty-eight hours post-transfection, the cells were lysed using Passive Lysis buffer (Promega) and luciferase activities were assessed using a dual luciferase reporter assay (Promega). The Renilla luciferase values were normalized to *firefly* luciferase values.

### Antibodies

Monoclonal mouse anti-FLAG M2 antibody (Sigma Aldrich, F1804, 1 mg ml^−1^), polyclonal rabbit anti-Flag antibody (Sigma Aldrich, F7425, 0.8 mg ml^−1^), monoclonal mouse anti-HA antibody (Sigma Aldrich, H3663, 1 mg ml^−1^) and polyclonal rabbit anti-HA antibody (Sigma Aldrich, H6908, 1 mg ml^−1^) were purchased from Sigma-Aldrich. All antibodies were used between 0.8 and 1 mg ml^−1^ for western blots.

### Structural figure generation and analysis

All structure analyses were carried out by various programs as implemented in the CCP4 program suite^30^. AREAIMOL and *S*c, respectively, were used for interaction surface analysis. Final structure figures were prepared using PyMOL^35^ and the protein–protein interactions were analysed using DIMPLOT as implemented in LigPlot^+^ (ref. 36).

### Data availability

Structures for eVP30/eNP complex have been deposited in the Protein Data Bank (http://www.rcsb.org) under accession codes 5VAP and 5VAO. All other relevant data are available from the authors upon request.

## Additional information

**How to cite this article:** Xu, W. *et al*. Ebola virus VP30 and nucleoprotein interactions modulate viral RNA synthesis. *Nat. Commun.* **8**, 15576 doi: 10.1038/ncomms15576 (2017).

**Publisher's note:** Springer Nature remains neutral with regard to jurisdictional claims in published maps and institutional affiliations.

## Supplementary Material

Supplementary Information Supplementary Figures and Supplementary Table

## Figures and Tables

**Table 1:** Data collection and refinement statistics.

**PDB ID**	**5VAP**	**5VAO**
*Data collection*		
Space group	P212121	I4122
Unit cell parameters		
*a*, *b*, *c* (Å)	48.5, 55.1, 114.6	139.8, 139.8, 161.7
*α*, *β*, *γ* (°)	90, 90, 90	90, 90, 90
Resolution range (Å)	50–1.85 (1.88–1.85)*	50–2.55 (2.59–2.55)
*R*_merge_ (%)	0.058 (0.636)	0.109 (0.982)
*I/σ*(*I*)	33.9 (2.1)	19.6 (1.0)
Completeness (%)	99.6 (100.0)	99.7 (94.9)
Redundancy	4.8 (4.8)	7.6 (4.3)
CC1/2	0.804	0.578
		
*Refinement*		
Resolution (Å)	57.31–1.85	105.55–2.56
No. of reflections	24,591	21,271
*R*_work_*/R*_free_ (%)	20.6/24.4	19.4/23.3
No. of atoms	2,123	4,353
Protein	1,991	4,318
Ligand/ion	24	7
Water	108	28
*B*-factors (Å^2^)		
Protein	20.840	52.882
	24.668	30.382
	35.248	49.045
	20.704	35.222
		32.509
		61.506
		39.597
		74.579
Ligand/ion	48.256	75.540
		59.350
Water	28.011	24.015
R.m.s.d.		
Bond lengths (Å)	0.023	0.015
Bond angles (°)	1.106	0.770
Ramachandran plot outliers (%)	0.00	0.00
MolProbity score	0.91	1.21
MolProbity clashscore	1.64	4.34

^*^Highest resolution shell is shown in parenthesis.

## References

[b1] Messaoudi I., Amarasinghe G. K. & Basler C. F. Filovirus pathogenesis and immune evasion: insights from Ebola virus and Marburg virus. Nat. Rev. Microbiol. 13, 663–676 (2015). 26439085 10.1038/nrmicro3524 PMC5201123

[b2] Misasi J. & Sullivan N. J. Camouflage and misdirection: the full-on assault of ebola virus disease. Cell 159, 477–486 (2014). 25417101 10.1016/j.cell.2014.10.006 PMC4243531

[b3] Kuhn J. H. . Filovirus RefSeq entries: evaluation and selection of filovirus type variants, type sequences, and names. Viruses 6, 3663–3682 (2014). 25256396 10.3390/v6093663 PMC4189044

[b4] Basler C. F. New hope in the search for Ebola virus treatments. Immunity 41, 515–517 (2014). 25367568 10.1016/j.immuni.2014.10.001

[b5] Feldmann H., Jones S. M., Schnittler H. J. & Geisbert T. Therapy and prophylaxis of Ebola virus infections. Curr. Opin. Investig. Drugs 6, 823–830 (2005). 16121689

[b6] Muhlberger E. Filovirus replication and transcription. Future Virol. 2, 205–215 (2007). 24093048 10.2217/17460794.2.2.205 PMC3787895

[b7] John S. P. . Ebola virus VP30 is an RNA binding protein. J. Virol. 81, 8967–8976 (2007). 17567691 10.1128/JVI.02523-06 PMC1951390

[b8] Modrof J., Becker S. & Muhlberger E. Ebola virus transcription activator VP30 is a zinc-binding protein. J. Virol. 77, 3334–3338 (2003). 12584359 10.1128/JVI.77.5.3334-3338.2003 PMC149768

[b9] Modrof J. . Phosphorylation of Marburg virus VP30 at serines 40 and 42 is critical for its interaction with NP inclusions. Virology 287, 171–182 (2001). 11504552 10.1006/viro.2001.1027

[b10] Modrof J., Muhlberger E., Klenk H. D. & Becker S. Phosphorylation of VP30 impairs Ebola virus transcription. J. Biol. Chem. 277, 33099–33104 (2002). 12052831 10.1074/jbc.M203775200

[b11] Martinez M. J. . Role of VP30 phosphorylation in the Ebola virus replication cycle. J. Infect. Dis. 204, S934–S940 (2011). 21987772 10.1093/infdis/jir320

[b12] Biedenkopf N., Hartlieb B., Hoenen T. & Becker S. Phosphorylation of Ebola virus VP30 influences the composition of the viral nucleocapsid complex: impact on viral transcription and replication. J. Biol. Chem. 288, 11165–11174 (2013). 23493393 10.1074/jbc.M113.461285 PMC3630872

[b13] Groseth A. . The Ebola virus ribonucleoprotein complex: a novel VP30-L interaction identified. Virus Res. 140, 8–14 (2009). 19041915 10.1016/j.virusres.2008.10.017 PMC3398801

[b14] Becker S., Rinne C., Hofsass U., Klenk H. D. & Muhlberger E. Interactions of Marburg virus nucleocapsid proteins. Virology 249, 406–417 (1998). 9791031 10.1006/viro.1998.9328

[b15] Hartlieb B., Muziol T., Weissenhorn W. & Becker S. Crystal structure of the C-terminal domain of Ebola virus VP30 reveals a role in transcription and nucleocapsid association. Proc. Natl Acad. Sci. USA 104, 624–629 (2007). 17202263 10.1073/pnas.0606730104 PMC2111399

[b16] Weik M., Modrof J., Klenk H. D., Becker S. & Muhlberger E. Ebola virus VP30-mediated transcription is regulated by RNA secondary structure formation. J. Virol. 76, 8532–8539 (2002). 12163572 10.1128/JVI.76.17.8532-8539.2002 PMC136988

[b17] Martinez M. J. . Role of Ebola virus VP30 in transcription reinitiation. J. Virol. 82, 12569–12573 (2008). 18829754 10.1128/JVI.01395-08 PMC2593317

[b18] Enterlein S. . Rescue of recombinant Marburg virus from cDNA is dependent on nucleocapsid protein VP30. J. Virol. 80, 1038–1043 (2006). 16379005 10.1128/JVI.80.2.1038-1043.2006 PMC1346851

[b19] Dziubanska P. J., Derewenda U., Ellena J. F., Engel D. A. & Derewenda Z. S. The structure of the C-terminal domain of the Zaire ebolavirus nucleoprotein. Acta Crystallogr. D Biol. Crystallogr. 70, 2420–2429 (2014). 25195755 10.1107/S1399004714014710 PMC4157450

[b20] Baker L. E. . Molecular architecture of the nucleoprotein C-terminal domain from the Ebola and Marburg viruses. Acta Crystallogr. D Struct. Biol. 72, 49–58 (2016). 26894534 10.1107/S2059798315021439 PMC4905509

[b21] Hartlieb B., Modrof J., Muhlberger E., Klenk H. D. & Becker S. Oligomerization of Ebola virus VP30 is essential for viral transcription and can be inhibited by a synthetic peptide. J. Biol. Chem. 278, 41830–41836 (2003). 12912982 10.1074/jbc.M307036200

[b22] Muhlberger E., Weik M., Volchkov V. E., Klenk H. D. & Becker S. Comparison of the transcription and replication strategies of marburg virus and Ebola virus by using artificial replication systems. J. Virol. 73, 2333–2342 (1999). 9971816 10.1128/jvi.73.3.2333-2342.1999 PMC104478

[b23] Kirchdoerfer R. N., Moyer C. L., Abelson D. M. & Saphire E. O. The Ebola virus VP30-NP interaction is a regulator of viral RNA synthesis. PLoS Pathog. 12, e1005937 (2016). 27755595 10.1371/journal.ppat.1005937 PMC5068707

[b24] Otwinowski Z. & Minor W. Processing of X-ray diffraction data collected in oscilation mode. Methods Enzymol. 276, 307–326 (1997). 10.1016/S0076-6879(97)76066-X 27754618

[b25] Minor W., Cymborowski M., Otwinowski Z. & Chruszcz M. HKL-3000: the integration of data reduction and structure solution--from diffraction images to an initial model in minutes. Acta Crystallogr. D Biol. Crystallogr. 62, 859–866 (2006). 16855301 10.1107/S0907444906019949

[b26] McCoy A. J. . Phaser crystallographic software. J. Appl. Crystallogr. 40, 658–674 (2007). 19461840 10.1107/S0021889807021206 PMC2483472

[b27] Cowtan K. The Buccaneer software for automated model building. 1. Tracing protein chains. Acta Crystallogr. D Biol. Crystallogr. 62, 1002–1011 (2006). 16929101 10.1107/S0907444906022116

[b28] Winn M. D. . Overview of the CCP4 suite and current developments. Acta Crystallogr. D Biol. Crystallogr. 67, 235–242 (2011). 21460441 10.1107/S0907444910045749 PMC3069738

[b29] Emsley P. & Cowtan K. Coot: model-building tools for molecular graphics. Acta Crystallogr. D Biol. Crystallogr. 60, 2126–2132 (2004). 15572765 10.1107/S0907444904019158

[b30] Collaborative Computational Project, N. The CCP4 suite: programs for protein crystallography. Acta Cryst. D50, 760–763 (1994). 10.1107/S0907444994003112 15299374

[b31] Davis I. W. . MolProbity: all-atom contacts and structure validation for proteins and nucleic acids. Nucleic Acids Res. 35, W375–W383 (2007). 17452350 10.1093/nar/gkm216 PMC1933162

[b32] Edwards M. R. . High-throughput minigenome system for identifying small-molecule inhibitors of Ebola virus replication. ACS Infect Dis. 1, 380–387 (2015). 26284260 10.1021/acsinfecdis.5b00053 PMC4537067

[b33] Hoenen M. R. . Oligomerization of Ebola virus VP40 is essential for particle morphogenesis and regulation of viral transcription. J Virol. 84, 7053–7063 (2010). 20463076 10.1128/JVI.00737-10 PMC2898221

[b34] Luthra P. . Mutual antagonism between the Ebola virus VP35 protein and the RIG-I activator PACT determines infection outcome. Cell Host Microbe. 14, 74–84 (2013). 23870315 10.1016/j.chom.2013.06.010 PMC3875338

[b35] DeLano W. L. The PyMOL Molecular Graphics System DeLano Scientific (2002).

[b36] Laskowski R. A. & Swindells M. B. LigPlot+: multiple ligand-protein interaction diagrams for drug discovery. J. Chem. Inf. Model 51, 2778–2786 (2011). 21919503 10.1021/ci200227u

