# Supplementary material for: Ebola virus VP30 and nucleoprotein interactions modulate viral RNA synthesis
Source: Nat Commun. 2017 Jun 8;8:15576. doi: 10.1038/ncomms15576 (PMC5472179; doi:10.1038/ncomms15576)
Supplement: Supplementary Information — Supplementary Figures and Supplementary Table [file ncomms15576-s1.pdf]

a

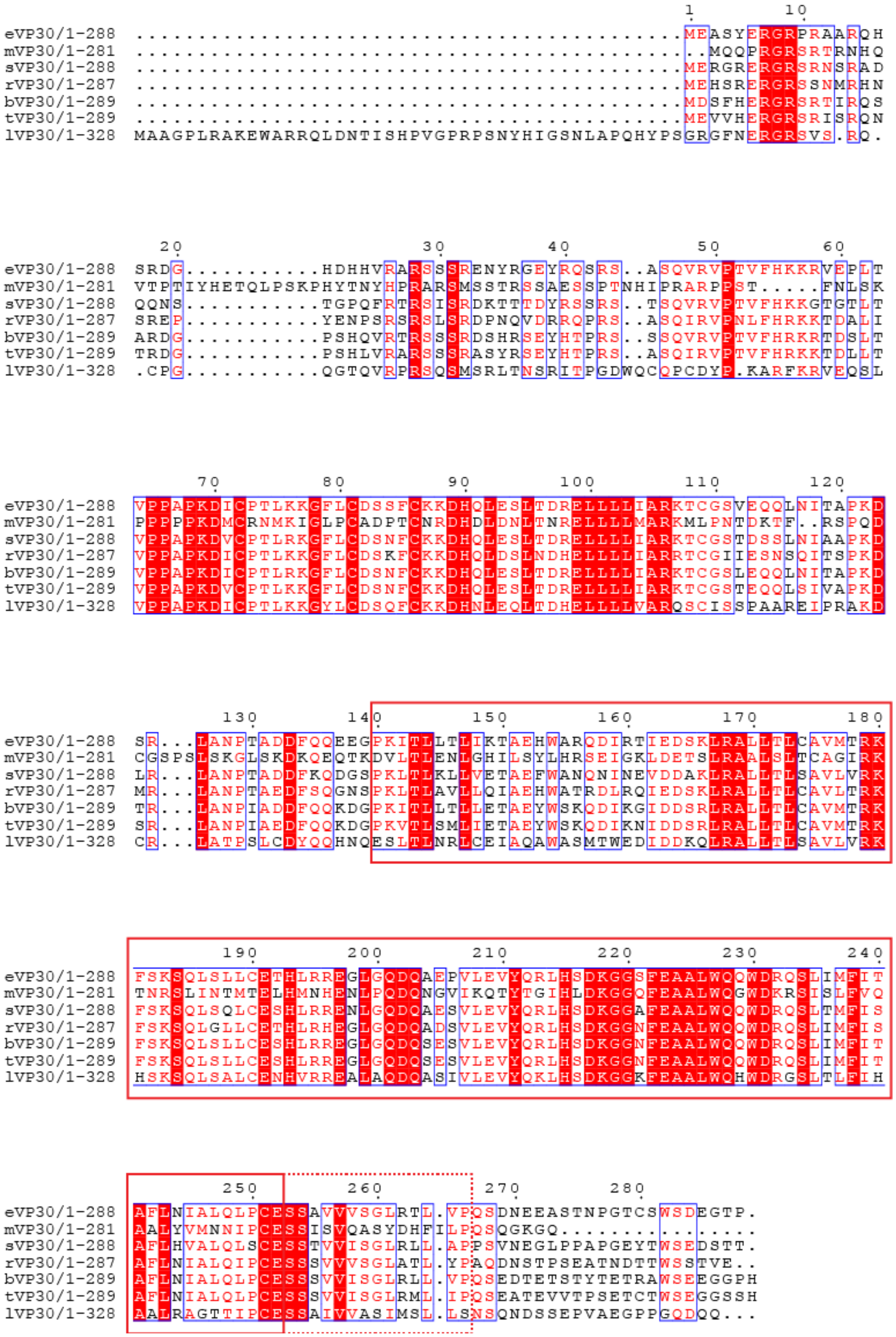

1 10
eVP30/1-288 ....MEASYERGRPRAARQH
mVP30/1-281 ....MQQPRGRSRTRNHQ
sVP30/1-288 ....MERGRERGRSRNSRAD
rVP30/1-287 ....MEHSRERGRSSNMRHN
bVP30/1-289 ....MDSFHERGRSRTIRQS
tVP30/1-289 ....MEVVHERGRSRISRQN
lVP30/1-328 MAAGPLRAKEWARRQLDNTISHPVGPRPSNYHIGSNLAPQHYPSGRGFNERGRSVS.RQ.
20 30 40 50 60
eVP30/1-288 SRDG...........HDHHVRARSSSRENYRGEYRQSRS..ASQVRVPTVFHKKRVEPLT
mVP30/1-281 VTPTIYHETQLPSKPHYTNYHPRARSMSSTRSSAESSPTNHIPRARPPST.....FNLSK
sVP30/1-288 QQNS...........TGPQFRTRSISRDKTTTDYRSSRS..TSQVRVPTVFHKKGTGTLT
rVP30/1-287 SREP...........YENPSRSRSLSRDPNQVDRRQPRS..ASQIRVPNLFHRKKTDALI
bVP30/1-289 ARDG...........PSHQVRTRSSSRDSHRSEYHTPRS..SSQVRVPTVFHRKRTDSLT
tVP30/1-289 TRDG...........PSHLVRARSSSRASYRSEYHTPRS..ASQIRVPTVFHRKKTDLLT
lVP30/1-328 .CPG...........QGTQVRPRSQSMSRLTNSRITPGDWQCQPCDYP.KARFKRVEQSL
70 80 90 100 110 120
eVP30/1-288 VPPAPKDICPTLKKGFLCDSSFCKKDHQLESLTDRELLLLIARKTCGSVEQQLNITAPKD
mVP30/1-281 PPPPPKDMCRNMKIGLPCADPTCNRDHDLDNLTNRELLLMARKMLPNTDKTF..RSPQD
sVP30/1-288 VPPAPKDVCPTLRKGFLCDSNFCKKDHQLESLTDRELLLLIARKTCGSTDSSLNIAAPKD
rVP30/1-287 VPPAPKDICPTLKKGFLCDSKFCKKDHQLDSLNDHELLLLIARRTCGIIESNSQITSPKD
bVP30/1-289 VPPAPKDICPTLRKGFLCDSNFCKKDHQLESLTDRELLLLIARKTCGSLEQQLNITAPKD
tVP30/1-289 VPPAPKDVCPTLKKGFLCDSNFCKKDHQLESLTDRELLLLIARKTCGSTEQQLSIVAPKD
lVP30/1-328 VPPAPKDICPTLKKGYLCDSQFCKKDHNLEQLTDHELLLLVARQSCISSPAAREIPRAKD
130 140 150 160 170 180
eVP30/1-288 SR...LANPTADDFQQEEGPKITLLTLIKTAEHWARQDIRTIEDSKLRALLTLCAVMTRK
mVP30/1-281 CGSPSLSKGLSKDKQEQTKDVLTLENLGHILSYLHRSEIGKLDETSLRAALSLTCAGIRK
sVP30/1-288 LR...LANPTADDFKQDGSPKLTLKLLVETAEFWANQNINEVDDAKLRALLTLSAVLVRK
rVP30/1-287 MR...LANPTAEDFSQGNSPKLTLAVLLQIAEHWATRDLRQIEDSKLRALLTLCAVLTRK
bVP30/1-289 TR...LANPIADDFQQKDGPKITLLTLLETAEYWSKQDIKGIDDSRLRALLTLCAVMTRK
tVP30/1-289 SR...LANPIAEDFQQKDGPKVTLSMLIETAEYWSKQDIKNIDDSRLRALLTLCAVMTRK
lVP30/1-328 CR...LATPSLCDYQQHNQESLTLNRLCEIAQAWASMTWEDIDDKQLRALLTLSAVLVRK
190 200 210 220 230 240
eVP30/1-288 FSKSQLSLLCETHLRREGLGQDQAEPVLEVYQRLHSDKGGSFEAALWQQWDRQSLIMFIT
mVP30/1-281 TNRSLINTMTELHMNHENLPQDQNGVIKQTYTGIHLDKGGQFEAALWQGWDKRSISLFVQ
sVP30/1-288 FSKSQLSQLCESHLRRENLGQDQAESVLEVYQRLHSDKGGAFEAALWQQWDRQSLTMFIS
rVP30/1-287 FSKSQLGLLCETHLRHEGLGQDQADSVLEVYQRLHSDKGGNFEAALWQQWDRQSLIMFIS
bVP30/1-289 FSKSQLSLLCESHLRREGLGQDQSESVLEVYQRLHSDKGGNFEAALWQQWDRQSLIMFIT
tVP30/1-289 FSKSQLSLLCESHLRREGLGQDQSESVLEVYQRLHSDKGGNFEAALWQQWDRQSLIMFIT
lVP30/1-328 HSKSQLSALCENHVRREALAQDQASIVLEVYQKLHSDKGGKFEAALWQHWDRGSLTLFIH
250 260 270 280
eVP30/1-288 AFLNIALQLPCESSAVVVSGLRTL.VPQSDNEEASTNPGTCSWSDEGTP.
mVP30/1-281 AALYVMNNIPCESSISVQASYDHFILPQSQGKGQ................
sVP30/1-288 AFLHVALQLSCESSTVVISGLRLL.APPSVNEGLPPAPGEYTWSEDSTT.
rVP30/1-287 AFLNIALQIPCESSSVVVSGLATL.YPAQDNSTPSEATNDTTWSSTVE..
bVP30/1-289 AFLNIALQLPCESSSVVISGLRLL.VPQSEDTETSTYTETRAWSEEGGPH
tVP30/1-289 AFLNIALQLPCESSSVVISGLRML.IPQSEATEVVTPSETCTWSEGGSSH
lVP30/1-328 AALRAGTTIPCESSAIVVASIMSL.LSNSQNDSSEPVAEGPPGQDQQ...

b

```
             1        10        20        30        40        50        60
eNP/1-739    MDSRPQKVWMTPSLTESDMDYHKILTAGLSVQQGIVRQRVIPVYQVNNLEEICQLIIQAF
mNP/1-695    ..................MDLHSLLELGTKPTAPHVRNKKVILFDTNHQVSICNQIIDAI
sNP/1-738    MDKRVRGSWALGGQSEVDLDYHKILTAGLSVQQGIVRQRVIPVYVVSDLEGICQHIIQAF
rNP/1-739    MDRGTRRIWVSQNQGDTDLDYHKILTAGLTVQQGIVRQKIISVYLVDNLEAMCQLVIQAF
bNP/1-739    MDPRPIRTWMMHNTSEVEADYHKILTAGLSVQQGIVRQRIIPVYQISNLEEVCQLIIQAF
tNP/1-739    MESRAHKAWMTHTASGFETDYHKILTAGLSVQQGIVRQRVIQVHQVTNLEEICQLIIQAF
lNP/1-749    MNRYLGHGTRTSRENTNLSELHGILSLGLNVDHTIVRKKSIPLFEIGNSDQVCNWIIQII

                      70        80        90       100       110       120
eNP/1-739    EAGVDFQESADSFLLMLCLHHAYQGDCKLFLESGAVKYLEGHGFRFEVKKRDGVKRLEEL
mNP/1-695    NSGIDLGDLLEGGLLTLCVEHYYNSDKDKFNTSPIAKYLRDAGYEFDVIKNADATRFLDV
sNP/1-738    EAGVDFQDNADSFLLLLCLHHAYQGDHRLFLKSDAVQYLEGHGFRFEVREKENVHRLDEL
rNP/1-739    EAGIDFQENADSFLLMLCLHHAYQGDYKLFLESNAVQYLEGHGFKFELRKKDGVNRLEEL
bNP/1-739    EAGVDFQDSADSFLLMLCLHHAYQGDYKQFLESNAVKYLEGHGFRFEMKKKEGVKRLEEL
tNP/1-739    EAGVDFQESADSFLLMLCLHHAYQGDYKQFLESNAVKYLEGHGFRFEVRKKEGVKRLEEL
lNP/1-749    EAGVDLQDVADSFLTMLCVNHAYQGDPNLFLESPAAHYLKGHGIHFEIQHRDNVDHITDL

                     130       140       150       160       170       180
eNP/1-739    LPAVSSGKNIKRTLAAMPEEETTEANAGQFLSFASLFLPKLVVGEKACLEKVQRQIQVHA
mNP/1-695    IPNEPHYSPLILALKTLESTESQRGRIGLFLSFCSLFLPKLVVGDRASIEKALRQVTVHQ
sNP/1-738    LPNVTGGKNLRRTLAAMPEEETTEANAGQFLSFASLFLPKLVVGEKACLEKVQRQIQVHA
rNP/1-739    LPAATSGKNIRRTLAALPEEETTEANAGQFLSFASLFLPKLVVGEKACLEKVQRQIQVHA
bNP/1-739    LPAASSGKNIKRTLAAMPEEETTEANAGQFLSFASLFLPKLVVGEKACLEKVQRQIQVHA
tNP/1-739    LPAASSGKSIRRTLAAMPEEETTEANAGQFLSFASLFLPKLVVGEKACLEKVQRQIQVHS
lNP/1-749    LGVGSRDKSLRKTLSALEFEPDGSTTAGMFLSFASLFLPKLVVGERACLEKVQRQIQIHA

                     190       200       210       220       230       240
eNP/1-739    EQGLIQYPTAWQSVGHMMVIFRLMRTNFLIKFLLIHQGMHMVAGHDANDAVISNSVAQAR
mNP/1-695    EQGIVTYPNHWLTTGHMKVIFGILRSSFILKFVLIHQGVNLVTGHDAYDSIISNSVGQTR
sNP/1-738    EQGLIQYPTSWQSVGHMMVIFRLMRTNFLIKFLLIHQGMHMVAGHDANDTVISNSVAQAR
rNP/1-739    EQGLIQYPTAWQSVGHMMVIFRLMRTNFLIKYLLIHQGMHMVAGHDANDAVIANSVAQAR
bNP/1-739    EQGLIQYPTSWQSVGHMMVIFRLMRTNFLIKFLLIHQGMHMVAGHDANDAVIANSVAQAR
tNP/1-739    EQGLIQYPTAWQSVGHMMVIFRLMRTNFLIKFLLIHQGMHMVAGHDANDAVIANSVAQAR
lNP/1-749    EQGLIQYPTQWQSVGHMMVVFRLIRVNFVLKFLLVHQGMHMMAGHDANDAIIANSISQTR

                     250       260       270       280       290       300
eNP/1-739    FSGLLIVKTVLDHILQKTERGVRLHPLARTAKVKNEVNSFKAALSSLAKHGEYAPFARLL
mNP/1-695    FSGLLIVKTVLEFILQKTDSGVTLHPLVRTSKVKNEVASFKQALSNLARHGEYAPFARVL
sNP/1-738    FSGLLIVKTVLDHILQKTDLGVRLHPLARTAKVKNEVSSFKAALGSLAKHGEYAPFARLL
rNP/1-739    FSGLLIVKTVLDHILQKTDQGVRLHPLARTAKVRNEVNAFKAALSSLAKHGEYAPFARLL
bNP/1-739    FSGLLIVKTVLDHILQKTEHGVRLHPLARTAKVKNEVSSFKAALASLAQHGEYAPFARLL
tNP/1-739    FSGLLIVKTVLDHILQKTEHGVRLHPLARTAKVKNEVNSFKAALSSLAQHGEYAPFARLL
lNP/1-749    FSGLLIVKTVLEHILQKTEAGVQLHPLARTSKVKGELLAFKSALEALASHREYAPFARLL

                     310       320       330       340       350       360
eNP/1-739    NLSGVNNLEHGLFPQLSAIALGVATAHGSTLAGVNVGEQYQQLREAATEAEKQLQQYAES
mNP/1-695    NLSGINNLEHGLYPQLSAIALGVATAHGSTLAGVNVGEQYQQLREAAHDAEVKLQRRHEH
sNP/1-738    NLSGVNNLEHGLYPQLSAIALGVATAHGSTLAGVNVGEQYQQLREAATEAEKQLQQYAET
rNP/1-739    NLSGVNNLEHGLYPQLSAIALGVATAHGSTLAGVNVGEQYQQLREAATEAEKQLQQYAES
bNP/1-739    NLSGVNNLEHGLFPQLSAIALGVATAHGSTLAGVNVGEQYQQLREAATEAEKQLQKYAES
tNP/1-739    NLSGVNNLEHGLFPQLSAIALGVATAHGSTLAGVNVGEQYQQLREAATEAEKQLQKYAES
lNP/1-749    NLSGVNNLEHGLYPQLSAIALGVATAHGSTLAGVNVSEQYQQLREAATEAEKQLQQHSEM

                     370       380       390       400       410       420
eNP/1-739    RELDHLGLDDQEKKILMNFHQKKNEISFQQTNAMVTLKKERLAKLTEAITAASLPKTSGH
mNP/1-695    QEIQAIAEDDEERKILEQFHLQKTEITHSQTLAVLSQKREKLARLAAEIENNIVEDQGFK
sNP/1-738    RELDNLGLDEQEKKILMSFHQKKNEISFQQTNAMVTLRKERLAKLTEAITTASKIKVGDR
rNP/1-739    RELDSLGLDDQERRILMNFHQKKNEISFQQTNAMVTLRKERLAKLTEAITLASRPNLGSR
bNP/1-739    RELDHLGLDDQEKKILKDFHQKKNEISFQQTTAMVTLRKERLAKLTEAITSTSILKTGRR
tNP/1-739    RELDHLGLDDQEKKILKDFHQKKNEISFQQTTAMVTLRKERLAKLTEAITSTSLLKTGKQ
lNP/1-749    RELETLGLDEQERKILATFHSRKNEINIQQTSSILAIRKERLRKLTEALNEEKNKNALDD
```

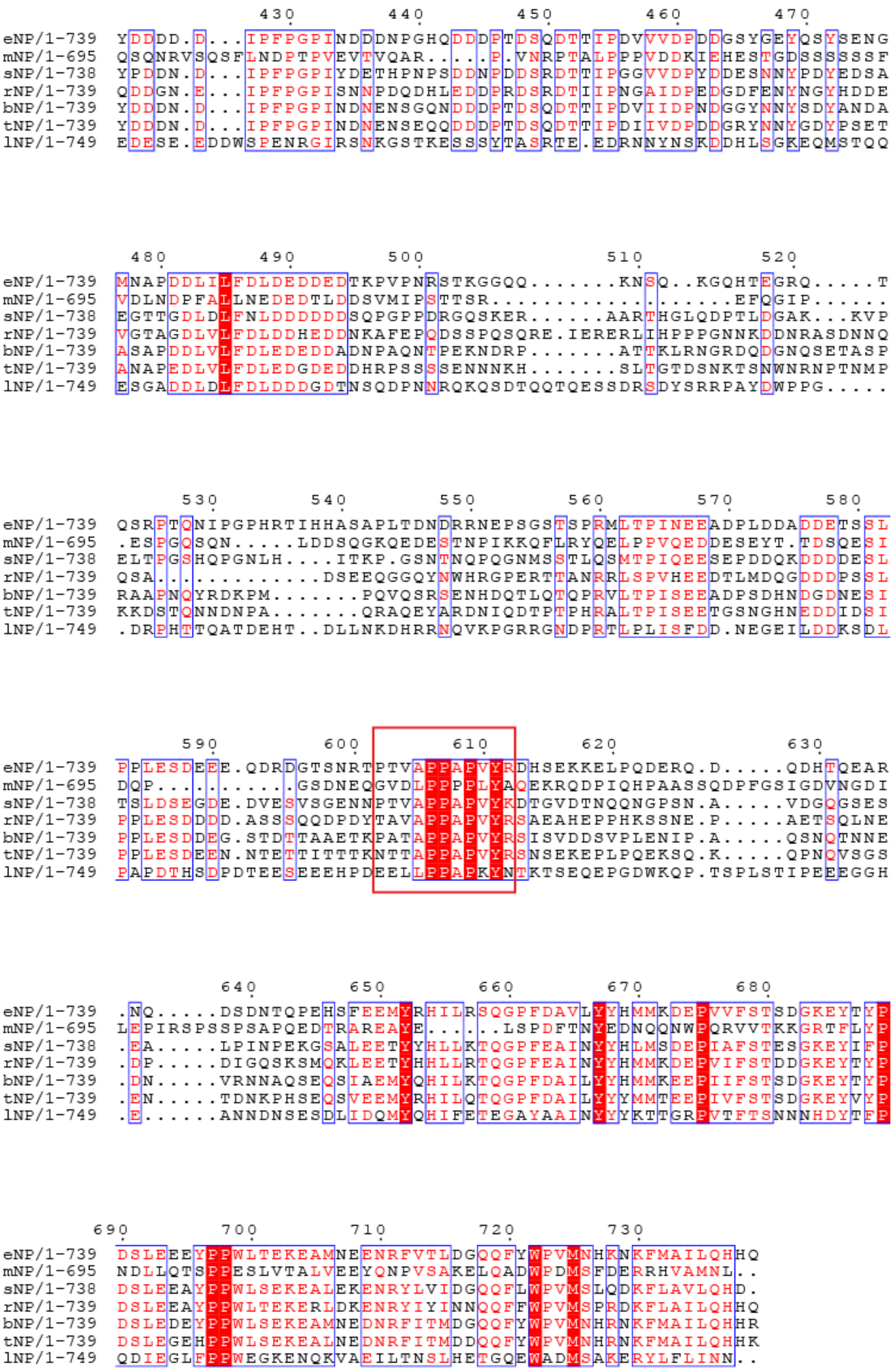

Supplementary Figure 1. Sequence alignment of filoviral NP and VP30 proteins. a, Sequences from Ebola virus VP30 (eVP30, Uniprot number: Q05323), Marburg virus VP30 (mVP30, Uniprot number: P35258), Sudan virus VP30 (sVP30, Uniprot number: Q5XX03), Reston virus VP30 (rVP30, Uniprot number: Q8JPX6), Bundibugyo virus

VP30 (bVP30, Uniprot number:B8XCN3), Tai Forest virus (tVP30, Uniprot number:B8XCP2), and Lloviu cuevavirus VP30 (lVP30, Uniprot number: G8EFI6) were aligned using CLUSTALO and colored by the percentage of identity. **b,** Sequences from Ebola virus NP (eNP, Uniprot number: L7QI51), Marburg virus NP (mNP, Uniprot number: C7B283), Sudan virus NP (sNP, Uiprot number: Q5XX08), Reston virus NP (rNP, Uniprot number: Q8JPY1), Bundibugyo virus NP (bNP, Uniprot number: R4QJ68), Tai Forest virus NP (tNP, Uniprot number:B8XCN6), and Lloviu cuevavirus NP (lNP, Uniprot number: G8EFI1) were aligned using CLUSTALO and colored by the percentage of identity eNP residues that bind to eVP30, along with the corresponding sequences from other filoviral NPs, are highlighted with a red line. eVP30 residues that bind to eNP peptide, along with the corresponding sequences from other filoviral VP30s, are highlighted with a red line for eVP30 residues 140-252 observed in all X-ray crystal structures and the dotted line represents additional residues 253-266 observed in PDB--4.

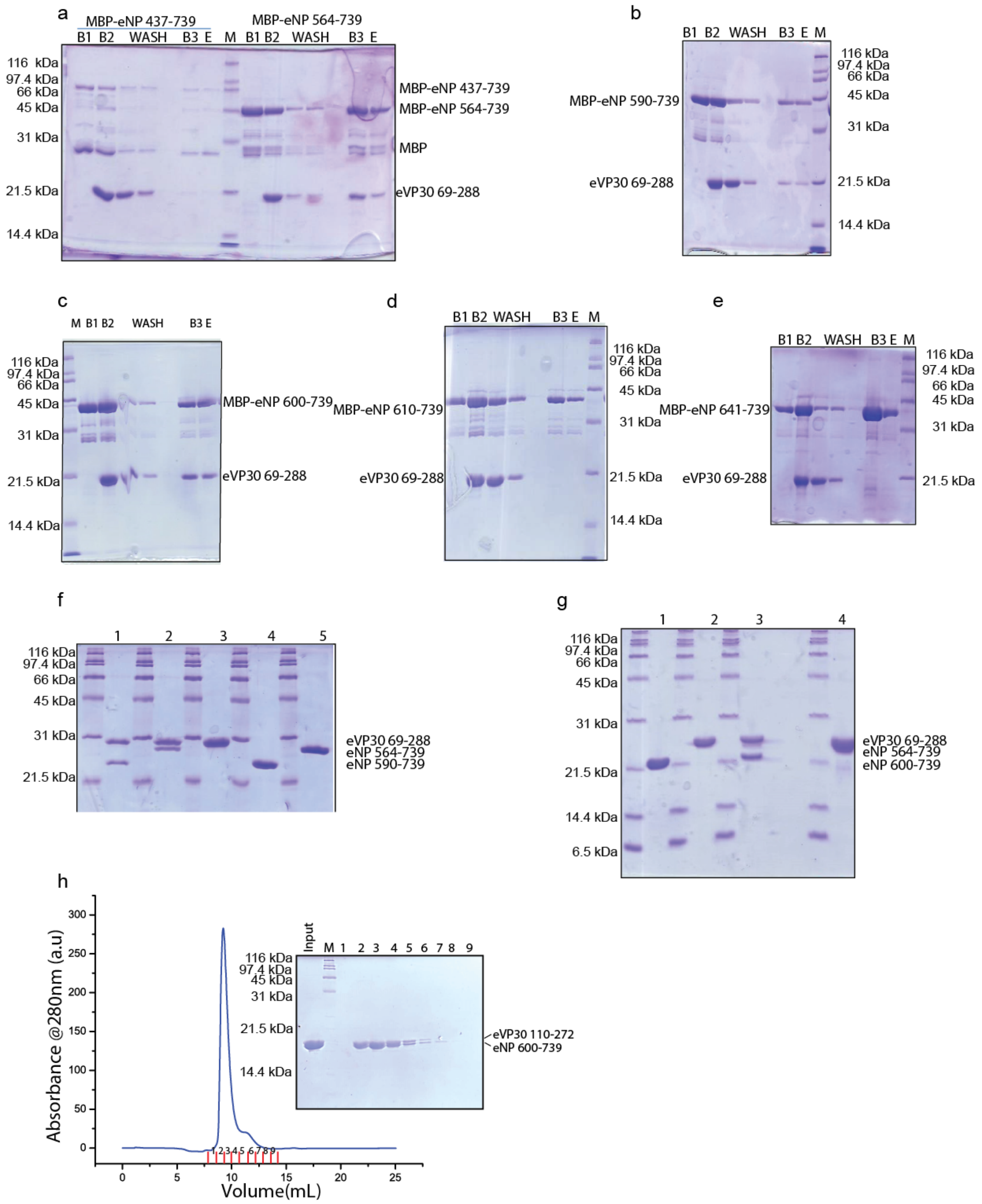

Supplementary Figure 2. eVP30 binds to a region of eNP that includes residues 600-612. Coomassie blue-stained SDS-PAGE gels of pulldown assays of: **a,** MBP-eNP$_{437-739}$ and eVP30$_{69-288}$, and MBP-eNP$_{564-739}$ and eVP30$_{69-288}$. **b,** MBP-eNP$_{\mathbf{590-739}}$ and

$eVP30_{69\text{-}288}$. **c,** MBP-$eNP_{600\text{-}739}$ and $eVP30_{69\text{-}288}$. **d,** MBP-$eNP_{610\text{-}739}$ and $eVP30_{69\text{-}288}$. **e,** MBP-$eNP_{641\text{-}739}$ and $eVP30_{69\text{-}288}$. **f,** Purified $eVP30_{69\text{-}288}$, $eNP_{564\text{-}739}$, $eNP_{590\text{-}739}$ and complex $eVP30_{69\text{-}288}$/$eNP_{590\text{-}739}$, $eVP30_{69\text{-}288}$/$eNP_{564\text{-}739}$. **g,** Purified $eVP30_{89\text{-}288}$, $eNP_{600\text{-}739}$ and complex $eVP30_{89\text{-}288}$/$eNP_{600\text{-}739}$, $eVP30_{89\text{-}288}$/$eNP_{564\text{-}739}$. **h,** Size exclusion chromatographic elution profile and corresponding SDS-PAGE of $eVP30_{110\text{-}272}$/$eNP_{600\text{-}739}$ complex. Markers are noted next to each gel panel.

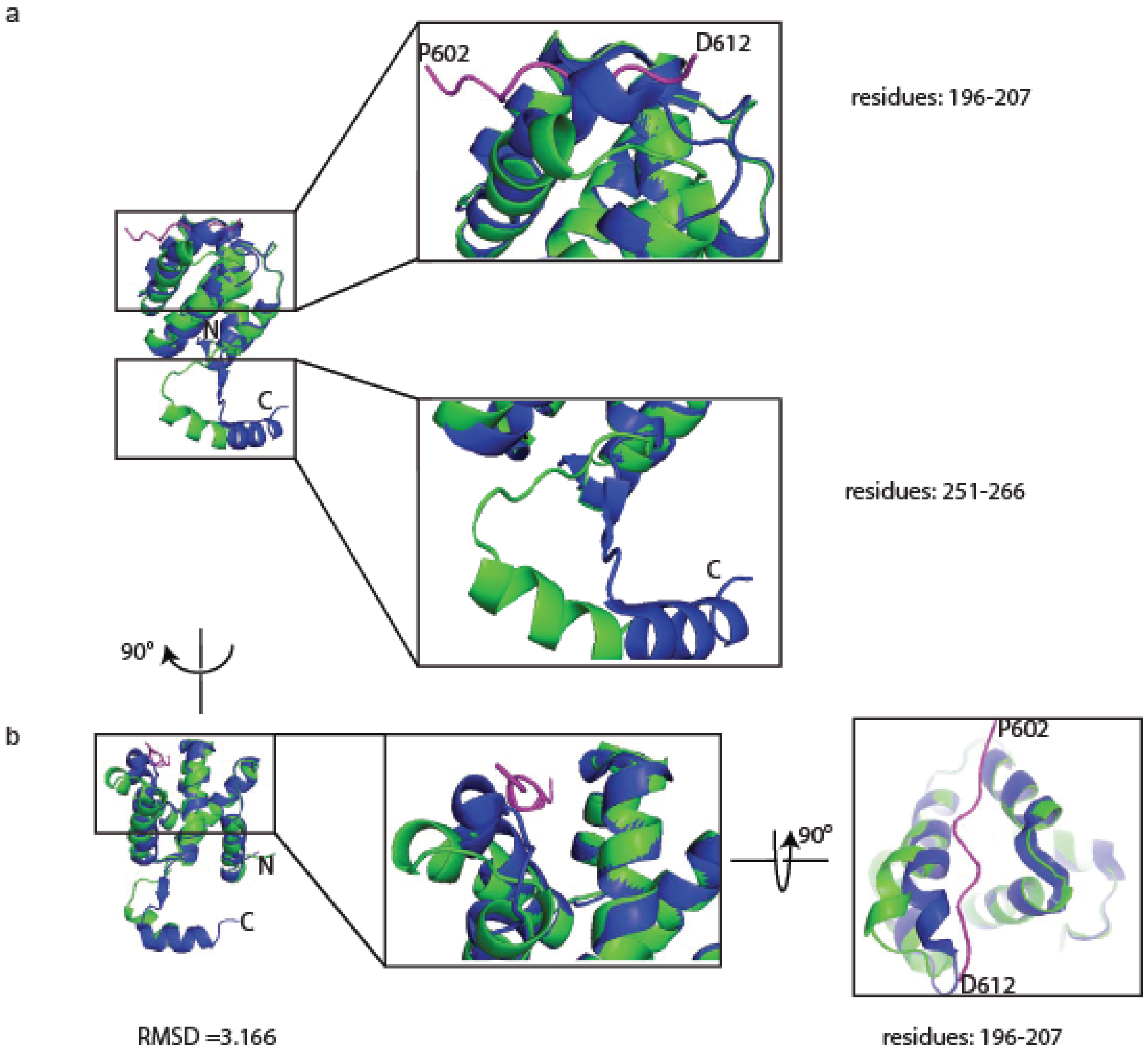

Supplementary Figure 3. Comparison of eVP30 structure alone and bound to eNP peptide. a, Overall structural alignment of structure from our study (green) with previously characterized free eVP30 (PDB 2I8B shown in blue; residues 142 to 266). Expansion to highlight regions corresponding to residue 196-207 and 251-266 are shown on the right. **b,** A 90 degree rotated view of the structures shown in panel **S3a**. Region corresponding residues 196-207 are shown on the expanded right panel.

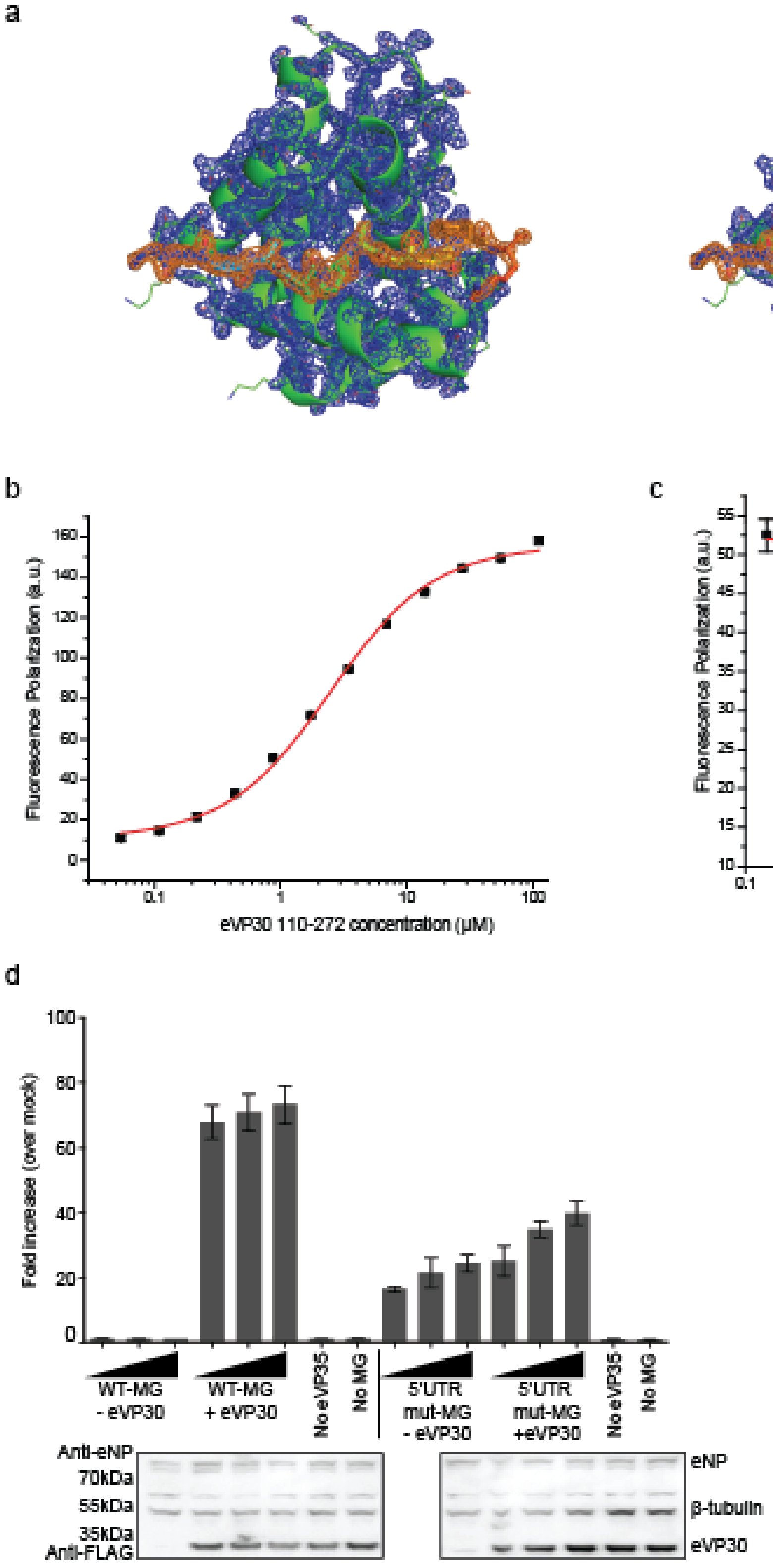
a
b
Fluorescence Polarization (a.u.)
eVP30 110-272 concentration (μM)
c
Fluorescence Polarization (a.u.)
eNP600-615 competitor concentration (μM)
d
Fold increase (over mock)
WT-MG
- eVP30
WT-MG
+ eVP30
No eVP35
No MG
5'UTR
mut-MG
- eVP30
5'UTR
mut-MG
+eVP30
No eVP35
No MG
Anti-eNP
70kDa
55kDa
35kDa
Anti-FLAG
eNP
β-tubulin
eVP30

Supplementary Figure 4. Optimization of fluorescence polarization assay for eVP30 binding to eNP peptide. a, Raw difference map of eVP30/eNP peptide complex showing initial unbiased electron density at 2σ. **b,** Raw polarization was measured with respect to increasing eVP30 protein concentrations (From 0.048 μM to 100 μM) for FITC-labeled $eNP_{600-615}$ peptide. **c,** Competition by unlabeled $eNP_{600-615}$ peptide. Increasing concentration of unlabeled competitor peptide $eNP_{600-615}$ peptide was used to measure raw polarization in the presence of 125 nM FITC-$eNP_{600-615}$ peptide and 1.00 μM $eVP30_{110-272}$ protein. Western blot analysis corresponding to minigenome assays in **d,** The minigenome assay using wt and mutant minigenome constructs that lacks the eVP30 binding loop. The experiment is representative of three independent experiments. The error bars represent standard deviation of three replicates. The western blot shows the expression of NP and VP30 using anti-NP and anti-Flag antibody respectively.

Supplementary Table 1. Summary of binding and minigenome studies.

	Binding to NP FPA	WT MG	WT MG+GFP NP600-615	Mutant MG	Mutant MG + GFP NP600-615
wt	+++	+++	-	+++	+++
E197A	-	++	+	+++	+++
D202A	n.d.	+++	++	+++	+++
Q203A	++	++	+	+++	+++
197/202/203AAA	-	++++	+++	++++	+++
Q229A	++	+++	++	+++	+++
W230A	-	-	-	+++	+++
Q229A/W230A	-	-	-	+++	+++

"+" and "-" are compared wt VP30 binding or MG activity
